# Proton pump inhibitor co-therapy in patients receiving non-vitamin K antagonist oral anticoagulants: current evidence, gastrointestinal bleeding prevention, and clinical considerations

**DOI:** 10.3389/fcvm.2026.1816099

**Published:** 2026-06-16

**Authors:** Dong-Hyeok Kim, Yeji Kim, Moon-Hyun Kim, Jeongmin Kang, Junbeom Park

**Affiliations:** 1 Division of Cardiology, Ewha Womans University Seoul Hospital, Seoul, Republic of Korea; 2 Division of Cardiology, Ewha Womans University Mokdong Hospital, Seoul, Republic of Korea

**Keywords:** atrial fibrillation (AF), gastroprotection, non-vitamin K antagonist oral anticoagulants (NOAC), pharmacoepidemiology, proton pump inhibitors (PPI), upper gastrointestinal bleeding (UGIB)

## Abstract

**Background:**

Non-vitamin K antagonist oral anticoagulants (NOACs) are widely prescribed for stroke prevention in atrial fibrillation (AF). Although they reduce intracranial hemorrhage compared with warfarin, gastrointestinal bleeding (GIB), particularly upper GIB (UGIB), remains a clinically significant complication. Proton pump inhibitors (PPIs) are frequently co-prescribed for gastroprotection, yet the available evidence remains largely observational and subject to methodological heterogeneity and confounding by indication.

**Evidence base and review methodology:**

We conducted a structured narrative review of nationwide cohort studies, multi-database analyses, randomized trials, and meta-analyses evaluating PPI co-therapy in NOAC-treated populations, with emphasis on clinically adjudicated UGIB outcomes and methodological considerations including confounding by indication and endpoint variability.

**Clinical evidence and implications for cardiac rhythmology practice:**

A large Korean nationwide cohort demonstrated that PPI co-therapy in NOAC-treated AF patients was associated with reduced risks of UGIB hospitalization (weighted HR 0.825) and transfusion-requiring UGIB (weighted HR 0.798), particularly in high-risk subgroups. A meta-analysis of approximately 1.97 million oral anticoagulant users reported lower odds of total and major GIB with PPI use (OR ∼0.67–0.68). Randomized evidence suggests biological plausibility for reduction of gastroduodenal bleeding but highlights endpoint specificity. However, heterogeneity across analytic designs underscores persistent residual confounding.

**Conclusions:**

PPI co-therapy may represent a clinically pragmatic strategy pending dedicated randomized evidence in high-risk NOAC-treated patients. A pragmatic, risk-stratified approach appears most appropriate while awaiting confirmation from dedicated randomized trials.

## 1 Introduction

Non-vitamin K antagonist oral anticoagulants (NOACs) have become the standard of care for stroke prevention in atrial fibrillation (AF) and for the treatment of venous thromboembolism because of their favorable pharmacokinetic profiles and improved safety compared with warfarin (13). Large nationwide comparative effectiveness analyses consistently demonstrate substantially lower rates of intracranial hemorrhage with NOACs while maintaining comparable or superior thromboembolic protection (13).

However, as intracranial bleeding has declined in the NOAC era, gastrointestinal bleeding (GIB)—particularly upper gastrointestinal bleeding (UGIB)—has emerged as the predominant bleeding phenotype in anticoagulated populations (1, 13). AF affects more than 30 million individuals worldwide, and its prevalence continues to rise with population aging (13). Within this epidemiologic context, even modest bleeding risks translate into substantial clinical and healthcare burden. Importantly, major gastrointestinal bleeding frequently results in temporary or permanent interruption of oral anticoagulation (1), and interruption of therapy has been associated with increased thromboembolic risk and mortality (1). Thus, strategies that reduce clinically significant UGIB may yield downstream benefit by preserving continuity of stroke prevention therapy.

Gastrointestinal safety profiles differ across individual NOAC agents. Population-based comparative effectiveness studies report relatively higher rates of gastrointestinal bleeding with rivaroxaban and comparatively lower rates with apixaban, underscoring clinically meaningful heterogeneity among agents (8, 13). Bleeding risk is further amplified by patient-level factors such as advanced age, prior peptic ulcer disease, Helicobacter pylori infection, elevated HAS-BLED scores, and concomitant use of antiplatelet agents or nonsteroidal anti-inflammatory drugs (9). In contemporary cardiac rhythm practice, these overlapping risk factors frequently coexist, complicating individualized risk–benefit assessment.

Proton pump inhibitors (PPIs) are commonly co-prescribed to mitigate gastrointestinal risk in patients receiving oral anticoagulation. Observational studies suggest that PPI co-therapy may reduce hospitalization for UGIB and severe bleeding events among NOAC-treated patients, particularly in high-risk subgroups (1, 5). A nationwide Korean cohort demonstrated lower risks of UGIB hospitalization and transfusion-requiring bleeding among AF patients receiving NOAC therapy with concomitant PPI use (5), and meta-analytic synthesis across nearly two million oral anticoagulant users reported lower odds of total and major gastrointestinal bleeding with PPI co-therapy (3). Nevertheless, most available data are observational and susceptible to confounding by indication, and findings have not been entirely consistent across analytic designs (6).

Given the widespread global use of NOACs, the clinical consequences of UGIB, and the uncertainty regarding the magnitude and generalizability of gastroprotective benefit, a balanced and methodologically rigorous synthesis of contemporary evidence is required. This review summarizes population-based cohort studies, meta-analytic findings, randomized data, and mechanistic considerations regarding PPI co-therapy in NOAC-treated patients. We further integrate these data into a pragmatic, risk-stratified framework tailored to contemporary cardiac rhythmology practice.

## 2 Evidence base and review methodology

This review provides a structured narrative synthesis of contemporary evidence evaluating proton pump inhibitor (PPI) co-therapy in patients receiving non-vitamin K antagonist oral anticoagulants (NOACs), with a primary focus on clinically relevant gastrointestinal bleeding outcomes. Given that the majority of available data derive from observational pharmacoepidemiologic studies, our objective was not to perform quantitative pooling but to critically appraise the magnitude, consistency, and methodological context of reported associations. Although this review was designed as a structured narrative synthesis rather than a formal quantitative systematic review, elements of the PRISMA 2020 framework were applied to enhance transparency and reproducibility of study selection.

A structured literature search was conducted using PubMed as the primary database covering January 2015 to February 2026 using predefined Boolean combinations of terms including (“non-vitamin K antagonist oral anticoagulant” OR “direct oral anticoagulant” OR “NOAC”) AND (“proton pump inhibitor” OR “PPI”) AND (“gastrointestinal bleeding” OR “upper gastrointestinal bleeding” OR “UGIB”) AND (“atrial fibrillation”). To enhance completeness, manual screening of reference lists, citation tracking, and additional relevant sources were also performed. Original cohort studies, meta-analyses, and randomized trials reporting clinically adjudicated gastrointestinal bleeding outcomes were considered. Reviews without primary data, case reports, and non-English publications were excluded.

**Figure 1:**
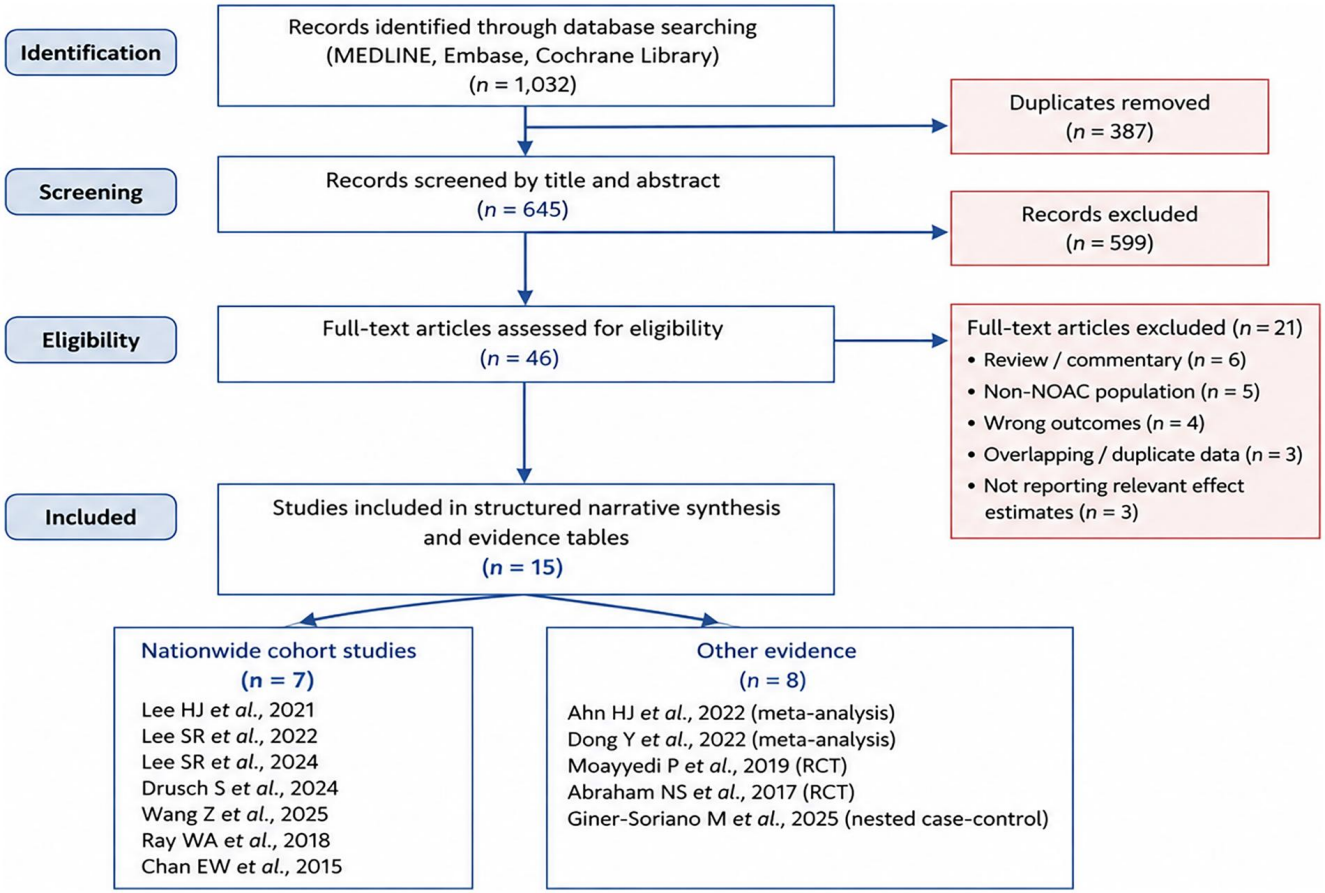
PRISMA flow diagram of study identification and selection process. The diagram illustrates the systematic identification, screening, eligibility assessment, and final inclusion of studies evaluating proton pump inhibitor (PPI) co-therapy in patients receiving non-vitamin K antagonist oral anticoagulants (NOACs). A total of 1,032 records were identified through database searching and other sources, of which 645 remained after duplicate removal. Following title and abstract screening, 46 full-text articles were assessed for eligibility, and 15 studies were ultimately included in the structured narrative synthesis, comprising nationwide cohort studies, multi-database analyses, meta-analyses, randomized trials, and other complementary evidence sources.

Study selection was performed through title and abstract screening followed by full-text evaluation. The study identification and selection process, including the number of records screened and reasons for exclusion, is summarized in Figure 1 in accordance with PRISMA reporting principles. Discrepancies in study eligibility were resolved through consensus discussion. Because this review was designed as a structured narrative synthesis rather than a quantitative meta-analysis, formal risk-of-bias scoring tools were not applied. Given the heterogeneity of included study designs, key bias domains were instead qualitatively evaluated and integrated into the interpretive discussion. These included confounding by indication, time-varying exposure, endpoint definition, and analytic framework.

Studies were prioritized if they reported clinically adjudicated outcomes, including upper gastrointestinal bleeding (UGIB), hospitalized gastrointestinal bleeding, or major gastrointestinal bleeding requiring transfusion or intervention. Particular attention was given to nationwide or multi-database cohort studies employing advanced confounding adjustment methods (e.g., propensity score weighting or matching), meta-analyses synthesizing large anticoagulated populations, and randomized trial data addressing acid suppression in anticoagulated patients.

**Figure 2:**
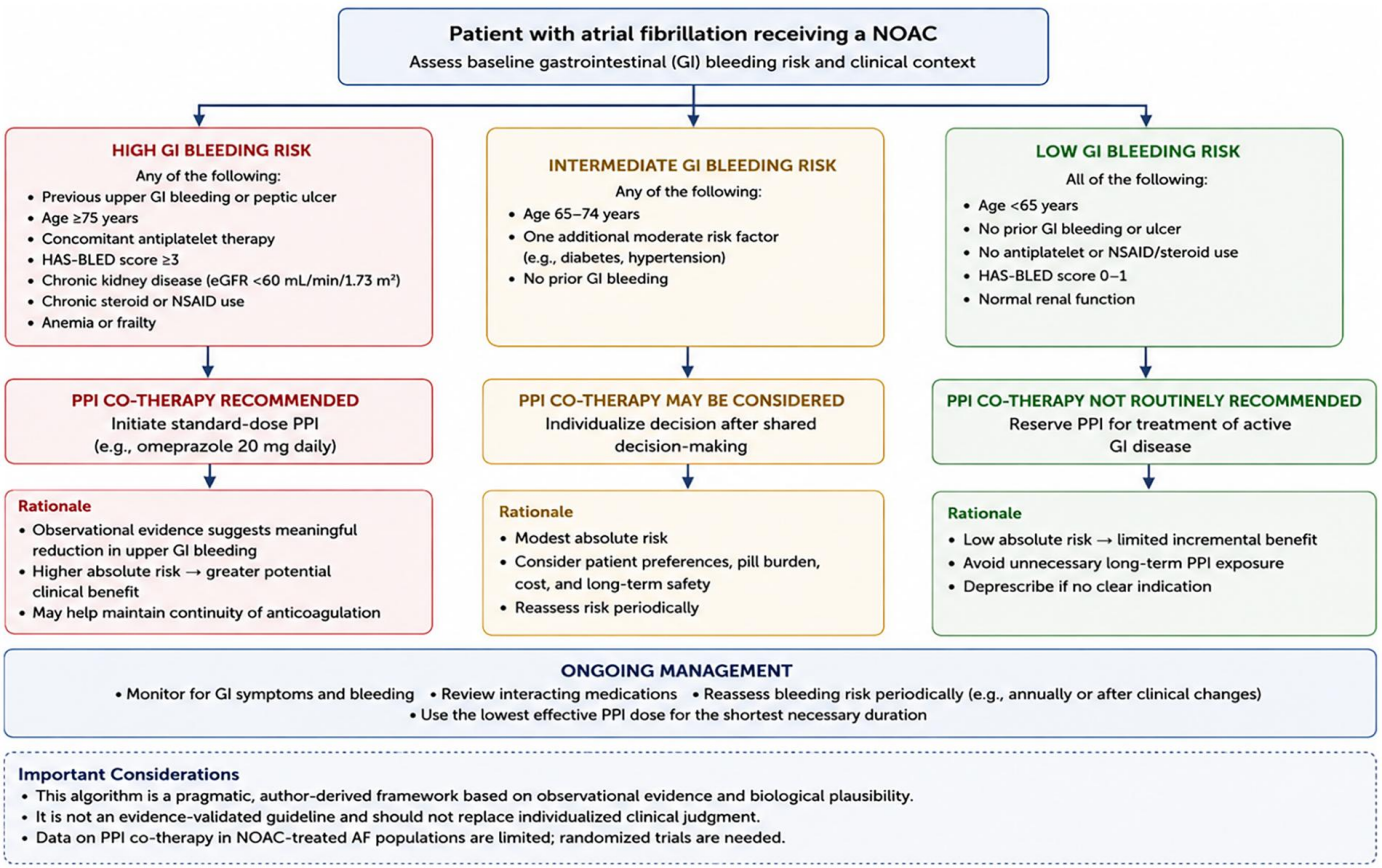
Conceptual clinical approach for proton pump inhibitor (PPI) co-therapy in patients receiving non-vitamin K antagonist oral anticoagulants (NOACs). The algorithm is informed by evidence from large population-based cohort studies and meta-analytic data summarized in Tables 1, 2 (2, 3, 5). It illustrates a pragmatic, risk-stratified conceptual approach to considering PPI co-therapy in patients treated with NOACs. The first step involves assessment of gastrointestinal (GI) bleeding risk, including advanced age (≥75 years), prior upper gastrointestinal bleeding (UGIB) or peptic ulcer disease, elevated bleeding risk scores (e.g., HAS-BLED ≥3), and concomitant use of antiplatelet agents, nonsteroidal anti-inflammatory drugs, or corticosteroids. In patients classified as having high GI bleeding risk, PPI co-therapy may be considered because observational studies have consistently demonstrated associations with lower rates of UGIB and related hospitalization. In patients with intermediate risk, decisions regarding PPI co-therapy may be individualized according to the overall balance of bleeding risk factors and clinical context. In patients with low GI bleeding risk and no prior GI pathology, the anticipated absolute benefit of routine long-term PPI co-therapy may be limited. Periodic reassessment of GI bleeding risk and ongoing need for PPI therapy is important, particularly during the early months following anticoagulant initiation and when concomitant medications or clinical risk profiles change. This approach seeks to balance the potential benefits of UGIB prevention against unnecessary long-term acid-suppression exposure. This figure represents an author-derived conceptual framework intended to illustrate pragmatic clinical considerations rather than an evidence-validated management pathway or formal clinical recommendation. NOAC, non-vitamin K antagonist oral anticoagulant; PPI, proton pump inhibitor; GI, gastrointestinal; UGIB, upper gastrointestinal bleeding.

Key characteristics and effect estimates from major cohort and meta-analytic investigations are summarized in Tables 1, 2. The integration of these data into a pragmatic, risk-stratified clinical framework is presented in Figure 2. This approach was designed to enhance transparency and reproducibility while maintaining the conceptual scope of a structured narrative review.

**Table 1:** Study characteristics of key studies included in the structured narrative review.

Study (year) *n* (populations)	Design data source	Population	Exposure comparison	Follow-up
Lee et al. (2021) (1) *n* = 19,851	Nationwide cohort (Korea NHIS)	AF patients receiving oral anticoagulant + PPI	NOAC vs. warfarin (all on PPI)	Mean ∼1.4 years
Lee et al. (2022) (2) *n* = 42,048	Cohort study (prior UGIB population)	AF patients on oral anticoagulants with prior UGIB	PPI vs. no PPI within anticoagulant strata	Not explicitly reported—described as moderate duration observational cohort
Ahn et al. (2022) (3) *n* = 1,970,931	Systematic review & meta-analysis (10 studies; ∼1.97M patients)	Oral anticoagulant users (NOAC and VKA)	PPI co-therapy vs. no PPI	Varied up to ∼5 years across included studies
Drusch et al. (2024) (4) *n* = 109,693	Nationwide cohort (France SNDS database)	Older AF patients initiating oral anticoagulants	PPI vs. no PPI	6 months and 12 months comparisons
Lee et al. (2024) (5) *n* = 65,756	Nationwide PS-weighted cohort (Korea HIRA)	AF patients receiving NOAC	PPI vs. no PPI	Median 1.5 years
Wang et al. (2025) (6) *n* = 343,451	PS-weighted cohort + case-crossover (England CPRD; Hong Kong CDARS)	AF oral anticoagulant users with NOAC subgroup	NOAC + PPI vs. NOAC only	Multi-year observational period
Giner-Soriano et al. (2025) (7) *n* = 28,504	Case-control (Catalonia SIDIAP database)	NVAF anticoagulated	PPI exposure among interacting drug users	3-month exposure window matched to event occurrence
Ray et al. (2018) (14) *n* = 1,643,123	Retrospective cohort study; US Medicare database	Patients receiving oral anticoagulants (apixaban, dabigatran, rivaroxaban, warfarin)	NOAC + PPI vs. NOAC only	Mean 264 days
Chan et al. (2015) (15) *n* = 5,041	Population-based retrospective cohort; Hong Kong Hospital Authority database	Newly prescribed dabigatran users	Dabigatran users receiving gastroprotective agents (primarily PPIs/H2RAs) vs. no gastroprotective therapy	Mean 7.2 months

AF, atrial fibrillation; UGIB, upper gastrointestinal bleeding; GI, gastrointestinal; PS, propensity score; NHIS, National Health Insurance Service; HIRA, Health Insurance Review and Assessment Service; SNDS, Système National des Données de Santé; CPRD, Clinical Practice Research Datalink; CDARS, Clinical Data Analysis and Reporting System; NVAF, nonvalvular atrial fibrillation.

**Table 2:** Key findings from major studies included in the structured narrative review.

Study (year) *n* (populations)	Primary bleeding outcome	Effect estimate	Summary interpretation
Lee et al. (2021) (1) *n* = 19,851	Upper GI bleeding (NOAC vs. warfarin in PPI users)	aHR 0.78 (95% CI 0.65–0.94)	Among patients receiving PPI co-therapy, NOAC use was associated with lower UGIB risk compared with warfarin
Lee et al. (2022) (2) *n* = 42,048	Major GI bleeding in patients with prior UGIB	Protective association observed in rivaroxaban and warfarin strata	Suggests potential benefit of PPI co-therapy in anticoagulated patients with previous UGIB
Ahn et al. (2022) (3) *n* = 1,970,931	Total and major GI bleeding (meta-analysis)	Total GIB: OR 0.67 (95% CI 0.62–0.74); Major GIB: OR 0.68 (95% CI 0.63–0.75)	PPI co-therapy associated with reduced GI bleeding risk across oral anticoagulant users
Drusch et al. (2024) (4) *n* = 109,693	Upper GI bleeding after OAC initiation	6 months: aHR 0.80 (95% CI 0.65–0.98); 12 months: aHR 0.90 (95% CI 0.76–1.07)	Early reduction in UGIB risk observed, with attenuation over time
Lee et al. (2024) (5) *n* = 65,756	Hospitalization for UGIB	wHR 0.825 (95% CI 0.761–0.894)	PPI co-therapy associated with lower risk of UGIB hospitalization, particularly in high-risk groups
Wang et al. (2025) (6) *n* = 343,451	Hospitalized GI bleeding	Cohort analysis: HR 1.23 (99% CI 1.02–1.44); Case-crossover: no significant modification effect	Cohort signal likely influenced by residual confounding; within-person design did not confirm modification.
Giner-Soriano et al. (2025) (7) *n* = 28,504	Major GI hemorrhage	OR 0.55 (95% CI 0.46–0.65)	PPI exposure associated with reduced GI hemorrhage risk; no protective association for cerebral hemorrhage
Ray et al. (2018) (14) *n* = 1,643,123	Retrospective cohort study; US Medicare database	PPI co-therapy associated with lower UGIB hospitalization risk	PPI co-therapy was associated with lower incidence of hospitalization for UGIB across OAC users; UGIB risk differed by anticoagulant type, with higher rates observed with rivaroxaban and lower rates with apixaban.
Chan et al. (2015) (15) *n* = 5,041	Population-based retrospective cohort; Hong Kong Hospital Authority database	Gastroprotective agents: IRR 0.52; PPI subgroup: IRR 0.53; UGIB: IRR 0.29	Gastroprotective agents were associated with lower GIB risk; the association was stronger for UGIB and in patients with prior peptic ulcer disease or GIB.

## 3 Discussion

### 3.1 Cohort evidence

Large nationwide cohort studies from East Asia and Europe provide consistent real-world signals suggesting an association between PPI co-therapy and reduced UGIB risk among NOAC-treated patients (1, 4, 5).

In a Korean nationwide cohort, PPI co-therapy was associated with lower risks of UGIB hospitalization (weighted HR 0.825) and transfusion-requiring UGIB (weighted HR 0.798), with more pronounced benefit in patients aged ≥75 years, those with HAS-BLED ≥3, prior UGIB, or concomitant antiplatelet therapy (5). Similar protective associations were observed in patients with a history of UGIB, supporting a role for secondary prevention strategies in selected populations (2).

European data add temporal nuance. In older patients initiating oral anticoagulants, PPI use was associated with reduced UGIB risk during the first 6 months, with attenuation of association at 12 months (4), suggesting that early treatment phases may represent periods of heightened gastrointestinal vulnerability.

However, multi-database analyses have demonstrated discordant findings depending on analytic design. In combined CPRD and CDARS datasets, propensity score–weighted cohort analyses and case-crossover approaches yielded differing conclusions, highlighting the influence of confounding by indication and methodological sensitivity (6).

While hazard ratios quantify relative risk reduction, clinical interpretation requires contextualization within baseline bleeding risk. In high-risk AF populations—particularly older patients and those receiving concomitant antiplatelet therapy—annual UGIB incidence may exceed 2%–3% (4, 5). In such groups, even modest relative risk reductions may translate into clinically meaningful absolute benefit. Conversely, in lower-risk individuals with baseline UGIB incidence below 1% (4), the absolute benefit of routine PPI co-therapy is likely small. As summarized in Table 1, variability in study design, population characteristics, and follow-up duration may partially explain differences in reported effect estimates across healthcare systems.

### 3.2 Meta-analytic data

A systematic review and meta-analysis including approximately 1.97 million oral anticoagulant users demonstrated that PPI co-therapy was associated with lower odds of total gastrointestinal bleeding (OR ∼0.67) and major gastrointestinal bleeding (OR ∼0.68) (3). These pooled estimates are broadly aligned with signals observed in nationwide cohort studies (1, 5), reinforcing a directionally consistent association between acid suppression and reduced gastrointestinal bleeding risk.

Importantly, however, pooled analyses also reveal agent-level variability among individual NOACs (3). Differences in intrinsic gastrointestinal bleeding risk—previously demonstrated in comparative effectiveness studies (8, 13)—suggest that baseline anticoagulant-specific risk profiles may influence the magnitude of observed benefit from PPI co-therapy. In other words, gastroprotective effect size is unlikely to be uniform across agents; rather, it may be amplified in populations receiving NOACs associated with relatively higher baseline gastrointestinal bleeding risk.

Because the majority of studies included in meta-analytic synthesis were observational, pooled associations may be interpreted as supportive rather than causal. Nevertheless, illustrative absolute risk modeling highlights clinical relevance. In populations with annual UGIB incidence approaching 3% (4, 5), a relative reduction of approximately 30% may translate into an absolute risk reduction near 1%, corresponding to an approximate NNT of 100 per year. These estimates are hypothesis-generating and intended to contextualize magnitude rather than imply causal effect and may not be interpreted as derived from pooled patient-level randomized data. In lower-risk populations with baseline risk near 1% (4), absolute benefit would be proportionally smaller. Thus, agent-level variability and baseline bleeding risk jointly determine the real-world clinical impact of PPI co-therapy. Key bleeding outcomes and effect estimates are summarized in Table 2.

### 3.3 Randomized evidence

Randomized evidence provides mechanistic support for a protective effect of acid suppression. In a double-blind trial evaluating pantoprazole in patients receiving rivaroxaban and/or aspirin, PPI therapy significantly reduced gastroduodenal bleeding events but did not significantly reduce a broader composite gastrointestinal endpoint (10). These findings suggest that gastroprotective benefit may be endpoint-specific and primarily confined to upper gastrointestinal pathology.

Mechanistically, PPIs promote stabilization of platelet-rich clots and facilitate mucosal healing in acid-mediated ulcer disease (9). Observational case-control data further demonstrate reduced gastrointestinal—but not intracranial—bleeding risk with PPI exposure, supporting anatomical specificity of protection (7).

From a clinical perspective, the concept of possible downstream clinical benefit is particularly relevant. Major gastrointestinal bleeding frequently necessitates interruption of anticoagulation therapy (1), which may increase thromboembolic risk. Therefore, prevention of clinically significant UGIB may indirectly preserve continuity of anticoagulation and support sustained stroke prevention in AF patients. Nevertheless, existing randomized trials were not specifically designed to evaluate PPI co-therapy exclusively in contemporary NOAC-treated atrial fibrillation cohorts. Consequently, while biologically coherent, definitive causal inference in this population remains incomplete. The contrast between observational and randomized findings is detailed in Table 2.

### 3.4 Heterogeneity and bias

Beyond agent-specific considerations, variability in reported associations across studies largely reflects methodological heterogeneity rather than biological inconsistency. Differences in population characteristics, endpoint definitions, follow-up duration, and analytic strategies materially influence observed effect estimates (1, 4, 6).

Confounding by indication remains central. Patients prescribed PPIs frequently exhibit higher baseline gastrointestinal risk, including prior ulcer disease, advanced age, and concomitant antiplatelet therapy (6, 9). Even with propensity score weighting or matching, residual confounding from unmeasured variables—such as Helicobacter pylori status, over-the-counter PPI use, medication adherence, and endoscopic severity—cannot be fully excluded (6, 9).

Additional unmeasured and partially measurable confounders may further influence the observed associations between PPI co-therapy and gastrointestinal outcomes. Frailty and competing mortality risk are particularly relevant in elderly anticoagulated populations, where patients perceived to be clinically vulnerable may be preferentially prescribed gastroprotective therapy while simultaneously exhibiting higher baseline risks of hospitalization and adverse outcomes independent of PPI exposure (6, 9, 14). Medication adherence to both NOAC and PPI therapy is also difficult to accurately ascertain in administrative datasets and may introduce healthy-adherer bias (6, 14). Furthermore, over-the-counter PPI use may result in exposure misclassification, potentially attenuating observed effect estimates (6, 15). Helicobacter pylori eradication status, socioeconomic disparities affecting healthcare utilization and endoscopic evaluation, and polypharmacy burden—including concomitant use of gastrotoxic or interacting medications—may additionally contribute to residual confounding not fully captured through conventional propensity adjustment methods (9, 14, 15). These limitations underscore the inherent challenges of causal inference in observational pharmacoepidemiologic analyses and support cautious interpretation of associative risk estimates.

Analytic design further shapes interpretation. Studies employing between-person cohort comparisons may yield different estimates than within-person case-crossover analyses, underscoring the sensitivity of findings to modeling framework (6). Additional epidemiologic biases should also be considered when interpreting observational associations between PPI co-therapy and gastrointestinal bleeding outcomes. Channeling bias may occur when clinicians preferentially prescribe PPIs to patients perceived to be at intrinsically higher gastrointestinal risk, thereby creating systematic differences between treated and untreated groups beyond measured covariates (6, 14). Surveillance bias may additionally influence outcome ascertainment, as patients receiving more intensive clinical follow-up or gastroenterologic evaluation may be more likely to undergo endoscopic investigation and bleeding detection. Furthermore, immortal time bias may arise in certain observational frameworks if periods preceding confirmed PPI exposure are incorrectly classified as exposed time, potentially exaggerating apparent protective associations (14). Although advanced analytic approaches, including time-dependent exposure modeling and propensity-based adjustment, may partially mitigate these limitations, residual confounding and bias cannot be fully eliminated in non-randomized pharmacoepidemiologic studies (6, 9, 14). These considerations further support cautious interpretation of associative effect estimates and highlight the need for dedicated randomized investigations. Time-varying exposure, reverse causation, and protopathic bias may additionally distort short-term associations when PPI initiation follows early gastrointestinal symptoms (6).

Thus, much of the observed variability across datasets may be understood as a function of methodological heterogeneity rather than contradictory biological signals. As summarized in Table 1, included studies range from nationwide propensity score–weighted cohorts to case-crossover designs, each with distinct strengths and limitations. Interpretation of effect magnitude must therefore account for study design and analytic structure alongside reported hazard ratios or odds ratios.

### 3.5 Pragmatic consideration

Taken together, the cumulative evidence suggests a structured, risk-stratified approach to PPI co-therapy in patients receiving NOACs rather than routine universal prophylaxis. Importantly, this recommendation is not based on clinical intuition alone but on reproducible observational signals across multiple healthcare systems (1, 2, 5).

First, clinicians may consider assessing baseline gastrointestinal bleeding risk using clinically accessible variables, including age ≥75 years, prior UGIB or peptic ulcer disease, HAS-BLED score ≥3, and concomitant antiplatelet or NSAID therapy (5, 9). Notably, observational cohort data demonstrate consistent protective associations of PPI co-therapy across datasets specifically within these higher-risk strata (1, 5). In particular, the magnitude of relative risk reduction appears more pronounced in patients with HAS-BLED ≥3, suggesting that gastroprotective benefit may scale with baseline bleeding vulnerability rather than being uniformly distributed across all AF populations (5).

Second, in patients with a documented history of UGIB, the signal for benefit is particularly robust. Independent analyses have demonstrated reduced recurrent major gastrointestinal bleeding in anticoagulated patients with prior UGIB receiving concomitant PPI therapy (2, 5). The replication of this secondary prevention signal across separate nationwide cohorts strengthens the plausibility of clinically meaningful benefit in this subgroup.

Third, anticoagulant selection may be integrated into individualized decision-making. Established differences in intrinsic gastrointestinal bleeding risk among NOAC agents—specifically higher rates with rivaroxaban and comparatively lower rates with apixaban—have been consistently demonstrated in large comparative effectiveness studies (8, 13). Consequently, the absolute risk reduction achievable with adjunctive PPI therapy is likely greater in patients treated with higher baseline bleeding–risk agents. In such cases, anticoagulant choice and gastroprotection may function as complementary strategies rather than independent considerations.

Fourth, gastrointestinal risk stratification may not be isolated from thromboembolic risk assessment. In atrial fibrillation management, bleeding and stroke risks frequently coexist. Major gastrointestinal bleeding commonly results in interruption of anticoagulation therapy (1), and interruption has been associated with increased thromboembolic events. Thus, selective prevention of clinically significant UGIB may indirectly preserve anticoagulation continuity and contribute to overall possible downstream clinical benefit in appropriately selected high-risk patients.

Finally, periodic reassessment remains essential. Gastrointestinal risk profiles evolve over time with changes in age, comorbidity burden, and concomitant medication exposure (4). Long-term PPI therapy may therefore be prescribed at the lowest effective dose and regularly re-evaluated, particularly in individuals with low anticipated absolute benefit and in light of potential long-term exposure considerations (11, 12).

This evidence-anchored, risk-stratified framework—summarized in Figure 2—emphasizes targeted implementation in high-risk patients while discouraging routine prophylaxis in low-risk individuals. The goal is not universal gastroprotection, but calibrated risk alignment grounded in reproducible cohort evidence and mechanistic plausibility.

### 3.6 Integrated interpretation

When the totality of evidence is considered, a coherent yet appropriately cautious interpretation emerges. Large population-based cohort studies consistently demonstrate associative signals suggesting reduced UGIB risk with PPI co-therapy in NOAC-treated patients, particularly in high-risk subgroups (1–3, 5). Meta-analytic synthesis further suggests directional consistency across populations (3), while randomized data provide mechanistic validation for upper gastrointestinal protection through acid suppression (10).

However, the current literature is characterized by a clear evidence hierarchy imbalance. The evidentiary base is weighted predominantly toward observational pharmacoepidemiologic studies, whereas randomized trials specifically designed to evaluate PPI co-therapy in contemporary NOAC-treated AF populations are lacking. Although several studies employed advanced adjustment methods to improve internal validity (6), interpretation of the available evidence remains constrained by the observational nature of the data (6, 9).

Importantly, the absence of definitive randomized confirmation does not negate biological plausibility. Rather, it delineates the boundary between associative consistency and causal certainty. Clinical impact is therefore highly contingent upon baseline bleeding risk. In high-risk patients, modest relative reductions may translate into meaningful absolute benefit and preservation of anticoagulation continuity. In contrast, in low-risk individuals, universal prophylaxis would likely yield limited incremental gain relative to medication burden.

Overall, convergence of epidemiologic consistency, agent-level biological rationale, and subgroup-specific signals suggests a calibrated, risk-stratified approach to PPI co-therapy. Such a strategy represents a clinically pragmatic and evidence-aligned response to an evidence base that is consistent in direction but incomplete in causal certainty.

## 4 Conclusions and future directions

PPI co-therapy appears associated with reduced UGIB risk among NOAC-treated patients, with greatest consistency in high-risk subgroups (2, 3, 5). However, methodological heterogeneity and residual confounding preclude definitive causal conclusions (6).

Several unresolved questions remain. First, the magnitude of absolute risk reduction achievable in clearly defined high-risk AF subgroups requires clarification (2, 5). Second, whether PPI co-therapy improves net clinical outcomes when ischemic stroke, bleeding, and mortality are considered jointly has not been formally tested in pragmatic randomized trials (1, 10). Third, effect modification by specific NOAC agents warrants further investigation (8, 13). Finally, long-term safety of chronic PPI exposure in elderly anticoagulated populations remains incompletely characterized (12).

Future pragmatic randomized trials incorporating stratified enrollment of high-risk patients and adjudicated time-to-event endpoints are needed to transition from associative evidence toward causal inference (6, 10). Optimizing UGIB prevention may ultimately support sustained anticoagulation adherence and improve long-term stroke prevention in atrial fibrillation.
